# The CLoCk study: A retrospective exploration of loneliness in children and young people during the COVID-19 pandemic, in England

**DOI:** 10.1371/journal.pone.0294165

**Published:** 2023-11-21

**Authors:** Kelsey McOwat, Snehal M. Pinto Pereira, Manjula D. Nugawela, Shamez N. Ladhani, Fiona Newlands, Terence Stephenson, Ruth Simmons, Malcolm G. Semple, Terry Segal, Marta Buszewicz, Isobel Heyman, Trudie Chalder, Tamsin Ford, Emma Dalrymple, Consortium, Roz Shafran

**Affiliations:** 1 Immunisations and Vaccine Preventable Diseases Department, UK Health Security Agency, London, United Kingdom; 2 Division of Surgery & Interventional Science, Faculty of Medical Sciences, University College London, London, United Kingdom; 3 University College London—Great Ormond Street Institute of Child Health, London, United Kingdom; 4 NIHR Health Protection Research Unit for Emerging and Zoonotic Infections, Institute of Infection, Veterinary and Ecological Sciences University of Liverpool, Liverpool, United Kingdom; 5 Respiratory Medicine, Alder Hey Children’s Hospital, Institute in The Park, University of Liverpool, Liverpool, United Kingdom; 6 Department of Paediatrics and Adolescence, University College London Hospital, London, United Kingdom; 7 Department of Psychological Medicine, Institute of Psychiatry, Psychology and Neuroscience, King’s College London, London, United Kingdom; 8 Department of Psychiatry, University of Cambridge, Hershel Smith Building Cambridge Biomedical Campus, Cambridge, United Kingdom; Oxford University Hospitals NHS Foundation Trust, UNITED KINGDOM

## Abstract

**Background:**

During the COVID-19 pandemic children and young people (CYP) were socially restricted during a stage of life crucial to development, potentially putting an already vulnerable population at higher risk of loneliness, social isolation, and poorer wellbeing. The objectives of this study are to conduct an exploratory analysis into loneliness before and during the pandemic, and determine which self-reported factors are associated with loneliness.

**Methods and findings:**

Participants from The Children with Long COVID (CLoCk) national study were invited to take part via an online survey, with a total of 31,017 participants taking part, 31,016 of which reported on their experience of loneliness. Participants retrospectively answered questions on demographics, lifestyle, physical health and mental health and loneliness before the pandemic and at the time of answering the survey. Before the pandemic 6.5% (2,006/31,016) of participants reported experiencing loneliness “Often/Always” and at the time of survey completion 17.4% (5,395/31,016) reported feeling lonelier. There was an association between meeting the research definition of long COVID and loneliness [3.49 OR, 95%CI 3.28–3.72]. CYP who reported feeling lonelier at the time of the survey than before the pandemic were assigned female at birth, older CYP, those from Black/African/Caribbean/Black British or other ethnicity groups, those that had 3–4 siblings and lived in more deprived areas.

**Conclusions:**

We demonstrate associations between multiple factors and experiences of loneliness during the pandemic. There is a need for a multi-faceted integrated approach when developing interventions targeted at loneliness. It is important to follow up the CYP involved at regular intervals to investigate the progression of their experience of loneliness over time.

## Introduction

The emergence of SARS-CoV-2 in December 2019 led to a global pandemic which saw most countries implement stringent infection prevention and control measures. These included social restrictions such as national lockdowns, where the majority of the population were required to stay at home with very limited exceptions. Children and young people (CYP) across the globe were restricted from meeting with peers and attending in-person education during a stage of life crucial for development, potentially putting an already vulnerable population at higher risk of loneliness, social isolation and exacerbating existing inequities [1–3].

Loneliness is defined as a subjective, negative, social and emotional state due to a perceived inconsistency between desired and achieved patterns of social contact, occurring when a person feels they have fewer social interactions than desired or that current social relationships lack quality characteristics such as available support [4, 5]. The definition of loneliness can be further broken down into social loneliness (the absence of social relationships) and emotional loneliness (the absence of close emotional attachment); together leading to feelings of boredom, exclusion, marginalisation, distress and apprehension [4]. Documented associated factors for feeling lonelier include social isolation, mental illness, being a victim of abuse and being a younger person or older adult (compared with middle-aged individuals) [4, 6, 7]. Loneliness is different to social Isolation, the latter is defined as a lack of social contacts and social relationships with regards to social network size, diversity and frequency [5, 8].

The Biopsychosocial Model of Health, developed by George Engel in 1977 explores the interactions between psychological, biological and social factors and how they influence an individual’s wellbeing, in this case, on the psychological experience of loneliness and vice versa [9]. Some studies have explored factors with a potential biological impact on loneliness such as age, sex at birth, genetics and family history of loneliness, some have explored the impact of psychological factors such as anxiety, while social impact studies have examined factors such as economic instability, social class and social restrictions [7, 10–13]. The relationship between experiences of loneliness and factors such as age, sex at birth, genetics and family history of loneliness is likely to be bi-directional [5, 8, 14, 15]. There is currently limited research on loneliness experienced during the pandemic in children, how their experiences of loneliness changed during the pandemic and which factors were associated with these changes.

The aim of this paper is to describe levels of loneliness before and during the pandemic and explore which factors relating to vulnerabilities and inequalities (e.g., pre-existing physical and mental health, ethnicity, index of multiple deprivation, age and sex at birth) are associated with different levels of loneliness. This exploration exploited validated scales such as the three-item UCLA loneliness scale (in addition to the one-item loneliness question), the Short Warwick Edinburgh Mental Wellbeing Scale (SWEMWBS), the Strengths and Difficulties questionnaire (SDQ) and the EQ-5D-Y in the CLoCk study. This manuscript is reported in accordance with the STROBE reporting guidelines from the EQUATOR network (S1 Appendix).

## Methods

### Participant sample and data collection

**Fig 1:**
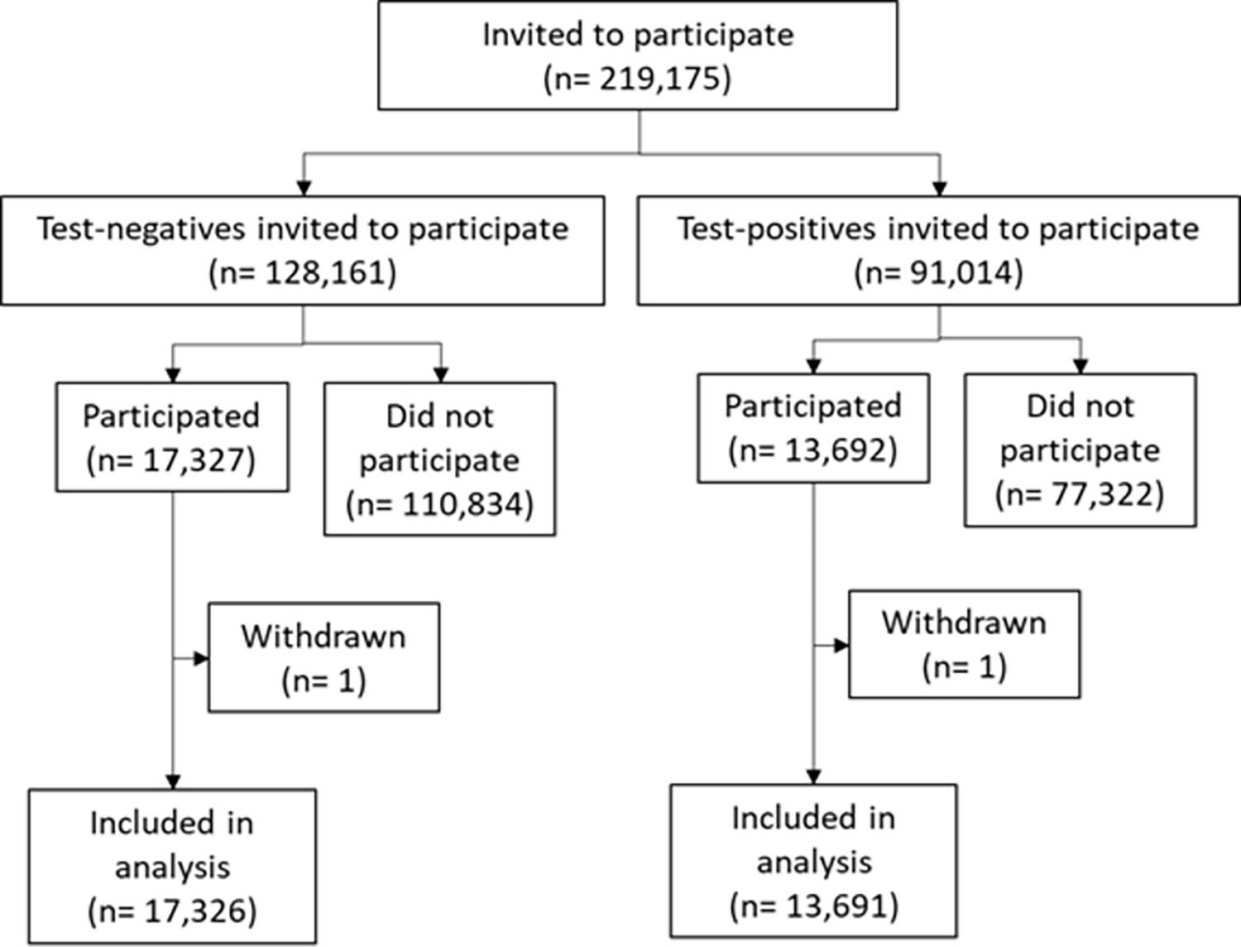
A flow chart of participation.

The CLoCk methodology has been published [16]. This is a national cohort study that invited a target population of 219,175 CYP who had a SARS-CoV-2 PCR positive or negative test between September 2020 and March 2021. Test positive CYP aged 11–17 years were identified through the national PCR-testing database held at UK Health Security Agency (UKHSA). They were matched to test negative CYP (matching on age, sex at birth and region) (Fig 1) [16]. CYP who had died, had no available address or had been involved in a previous study were excluded from the target population. 31,017 CYP that met the inclusion criteria and were initially tested between September 2020 and March 2021 took part in the study either 3 months, 6 months or 12 months after their test (Table 1). Initially a small proportion of CYP that were tested in December 2020 were invited and took part in the study due to funding constraints with more participants being invited at a later date. Participants were mailed an invitation letter to take part in the study via an online survey, answering questions on demographics, lifestyle, physical and mental health, both before the pandemic and at the time of the survey.

**Table 1:** Table of when participants were tested for COVID-19 and when they were invited to take part in the study.

Month COVID-19 test taken	Month invited to participate	Time between testing and study enrolment
September 2020	September 2021	12 months
October 2020	April 2021	6 months
November 2020	May 2021	6 months
December 2020 (part 1)	June 2021	6 months
December 2020 (part 2)	December 2021	12 months
January 2021	April 2021	3 months
February 2021	May 2021	3 months
March 2021	June 2021	3 months

### Ethical considerations

This survey collected written consent from participants aged 16 and over, or from the parent or guardian of children aged 11 to 15. Ethics approval was granted by Yorkshire & The Humber—South Yorkshire Research Ethics Committee (REC reference: 21/YH/0060; IRAS project ID:293495)

### Measures

At enrolment CYP were asked to retrospectively report on their feelings of loneliness before the pandemic as well as their experience of loneliness at the time of completing the survey. The 3-item UCLA loneliness scale assessed loneliness with higher total scores indicating a greater degree of experienced loneliness. The 3-item loneliness scale asks “How often do you feel that you have no one to talk to?”, “How often do you feel left out?” and “How often do you feel alone?”, with three response categories (1 = “Hardly ever or never”, 2 = “Some of the time” and 3 = “Often”) [17]. In addition to this, a single item loneliness question was asked: “How often do you feel lonely”, with 5 response categories (5 = “Often/Always”, 4 = “Some of the time”, 3 = “Occasionally”, 2 = “Hardly ever” and 1 = “Never”) [17]. As loneliness cannot be clinically diagnosed, there is no established cut-off on either scale to define loneliness [18]. Therefore, to evaluate change in loneliness, scores were categorised as “Less lonely”, “Same levels of loneliness” or “Lonelier” based on how their scores changed pre-pandemic to time of completing the survey. CYP were also asked to report on lifestyle and social factors such as days missed from school, and physical and mental health conditions including clinical vulnerabilities, such as respiratory conditions, that put individuals at increased risk of severe infection [19].

The Short Warwick Edinburgh Mental Wellbeing Scale (SWEMWBS) consists of 7 items, with reference to the 2 weeks prior to completing the survey with 5 response categories (1 = “None of the time”, 2 = “Rarely”, 3 = “Some of the time”, 4 = “Often” and 5 = “All of the time”). The scores for each of the 7 items were added together and then converted into metric scores with higher scores indicating better mental wellbeing [20].

The Strengths and Difficulties questionnaire (SDQ) use 25 items combined to form 5 subscales to assess emotional symptoms, conduct problems, hyperactivity-inattention, peer problems and prosocial behaviour [21, 22]. Each item is scored from 0 to 2, giving a score for each subscale ranging from 0 to 10. All the subscales, except the prosocial subscale, are summed to produce a total difficulties score ranging from 0 to 40. The SDQ also includes an Impact subscale to measure the impact of difficulties due to emotional and behavioural problems with a scoring of 0 to 10 with increasing impact producing a higher score [21, 22]. We used the following established cut-off points; > = 18 (total difficulties); > = 6 (emotional symptoms), > = 5 (conduct problems), > = 7 (hyperactivity); > = 4 (peer difficulties); < = 5 (prosocial skills) and > = 2 for impact [22]. The responses to the SDQ and the Impact score were each categorised into “Normal”, “Borderline” and “Abnormal”.

The EQ-5D-Y (developed by the EuroQol group to describe and value health-related quality of life) assessed participants health before the pandemic and at the time of completing the survey based on five dimensions; mobility, self-care, doing usual activities, experiencing pain/discomfort and anxiety/depression [23]. There were 3 response categories in relation to these dimensions: no problems, some problems, and a lot of problems.

As a proxy for socioeconomic status the Index of Multiple Deprivation (IMD) was derived from the CYPs lower super output area (LSOA; a small local area level-based geographical hierarchy), with quintiles from most (quintile 1) to least (quintile 5) deprived [16].

The operationalised Delphi research definition of long COVID in CYP is in alignment with the WHO clinical definition and states that “Post-COVID-19 condition occurs in young people with a history of confirmed SARS-CoV-2 infection, with one or more persisting physical symptoms for a minimum duration of 12 weeks after initial testing that cannot be explained by an alternative diagnosis. The symptoms have an impact on everyday functioning, may continue or develop after COVID-19 infection, and may fluctuate or relapse over time” [24, 25]. We operationalised this definition by a CYP having at least 1 symptom and experiencing some/ a lot of problems with respect to mobility, self-care, doing usual activities or having pain/discomfort or feeling very worried/sad [24]. Both test-positive and test-negative CYP may have met this definition.

### Statistical analysis

Data was analysed using STATA SE 17. The loneliness 3-item scale and single direct measure were calculated into separate binary variables with 0 = “No change/ Less lonely”, including those who experienced the same level or less loneliness over time, and 1 = “Lonelier”, including those who were lonelier at the time of the survey than before the pandemic. To explore the association, logistic regression was used to produce odds ratios (OR) for feeling lonelier, by the factors listed above. Relative risk (RR) was calculated by Poisson regression with robust standard errors.

## Results

### Participant demographics

A total of 219,175 CYP were invited to take part in the study and 31,017 enrolled (14.2%). 44.1% (13,691/31,017) tested SARS-CoV-2 PCR positive and 55.9% (17,326/31,017) tested negative at time of sampling (Fig 1). 61.4% (19,054/31,017) of participants were female and 38.6% (11,963/31,017) were male. 74.8% (23,201/31,017) of the participants identified as of White ethnicity, 14.7% (4,554/31,017) as Asian/Asian British, 5.2% (1,616/31,017) as Mixed, 3.0% (933/31,071) as Black/African/Caribbean/Black British, 1.7% (524/31,017) as Other, and 0.6% (189/31,017) preferred not to report their ethnicity. Of the 30,935 participants who reported on the number of siblings, 71.3% (22,062/30,935) had 1 or 2 siblings. A further breakdown of the target population matching demographics and analytical sample reported demographics (including ethnicity and number of siblings) are presented in Table 2.

**Table 2:** Table of target populations vs analytical sample demographics.

	Analytical sample n (%)
**Age (years)**	** **
11	2,934 (9.46)
12	3,548 (11.44)
13	3,992 (12.87)
14	4,386 (14.14)
15	5,596 (18.04)
16	5,342 (17.22)
17	5,219 (16.83)
**Sex at Birth**	** **
Female	19,054 (61.43)
Male	11,963 (38.57)
**Region**	** **
East Midlands	2,210 (7.13)
East of England	6,050 (19.51)
London	6,157 (19.85)
North East	1,198 (3.86)
North West	3,607 (11.63)
South East	4,917 (15.85)
South West	1,514 (4.88)
West Midlands	3,033 (9.78)
Yorkshire and The Humber	2,331 (7.52)
**Test result**	** **
Negative	17,326 (55.86)
Positive	13,691 (44.14)
**Ethnicity**	** **
Asian/Asian British	4,554 (14.68)
Black/African/Caribbean/Black British	933 (3.01)
Mixed	1,616 (5.21)
Other	524 (1.69)
Prefer not to say	189 (0.61)
White	23,201 (74.80)
**No. of Siblings**	** **
Only Child	2,741 (8.86)
1–2 siblings	22,062 (71.32)
3–4 siblings	4,976 (16.09)
5 or more siblings	1,156 (3.74)

### Pre-pandemic health

Of the 31,017 participants who reported on their physical health in general before the pandemic, 78.0% (24,179/31,017) reported Good or Very Good health, 20.2% (6,280/31,017) reported OK health and 1.8% (558/31,017) reported Poor or Very Poor health. Of the 31,017 participants who reported on their mental health in general before the pandemic 62.2% (19,284/31,017) reported Good or Very Good health, 29.1% (9,029/31,017) reported OK health and 8.7% (2,706/31,017) reported Poor or Very Poor health. When using the health scale of 0–100 (with 100 indicating best health) 64.3% of participants (19,942/31,016), chose a value of 90 or above on the scale.

When exploring clinical vulnerability and pre-existing health conditions, the most commonly reported conditions included tiredness (39.0%, 12,002/31,017), worry (36.9%, 11,440/31,017), and having an allergy (29.9%, 9,271/31,017). Only 7.4% of participants (2,300/31,017) reported taking prescribed medicine and 10.2% (3,173/31,017) reported receiving help for their mental health, while 1.8% (548/31,017) and 1.2% (364/31,017) of participants reported smoking or using e-cigarettes, respectively.

### Pre-pandemic vs current loneliness

Of the 31,017 participants that took part, 31,016 reported on their experience of loneliness. Before the pandemic more than half of participants reported “Never” or “Hardly ever” having experienced loneliness (28.6% and 30.6% respectively) with just 6.5% (2,006/31,016) reporting having “Often/Always” experienced loneliness. Descriptive analysis shows that before the pandemic those more likely to feel lonely “Often/Always” were older, female, had more siblings and lived in more deprived areas (S1 Table).

**Fig 2:**
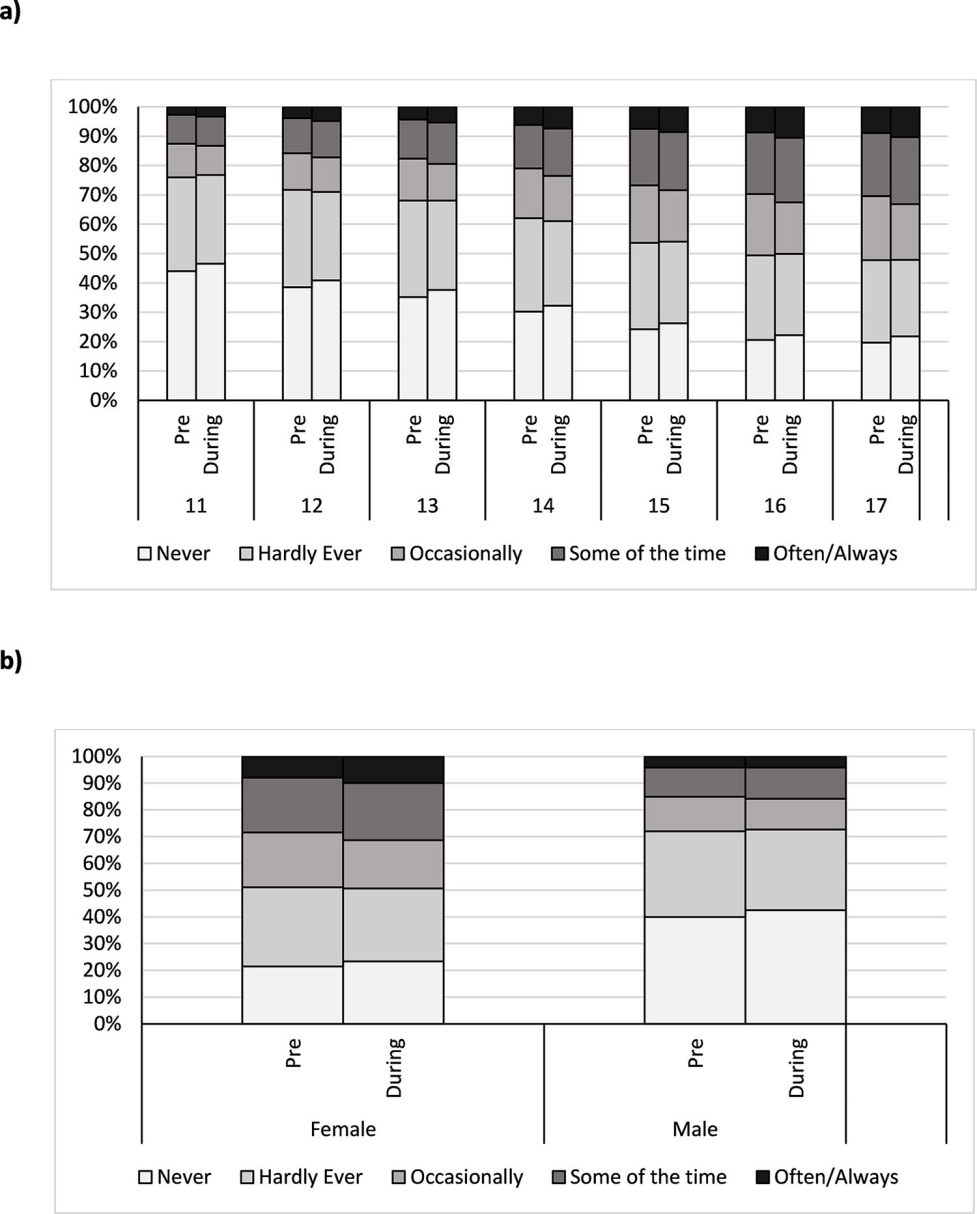
Graph of proportion of loneliness experienced before and during the pandemic against demographics a) age at time of test and b) sex at birth, using the one-item loneliness scale.

Of the 31,016 study participants reporting on loneliness using the 3-item loneliness scale, 63.7% (19,754/31,016) experienced the same frequency of loneliness before the pandemic and at the time of survey completion. 17.4% (5,395/31,016) reported feeling lonelier at the time of completing the survey in comparison to before the pandemic, while 18.9% (5,867/31,016) experienced the feeling of loneliness less at the time of completing the survey than before the pandemic (Fig 2, S2 Table and S1 Fig).

The majority of CYP in our analytical sample experienced the same level of loneliness before the pandemic and at the time of completing the survey when assessed using both the single direct loneliness measure and the 3-item loneliness scale (66.9% and 63.7% respectively). When asked “how often do you feel lonely?” 83.5% (25,905/31,016) of participants felt the same level of loneliness or felt less lonely at the time of survey completion in comparison to before the pandemic, while 16.5% (5,111/31,016) felt lonelier over the course of the pandemic. The difference in experienced loneliness before the pandemic and at the time of survey completion showed that a majority of those that reported a change in their experienced loneliness, reported one increment of change 22.1% (6,856/31,016), for example their frequency of experienced loneliness changed from “Often/Always” to “Some of the time” or vice versa.

### Factors associated with loneliness during the pandemic

Descriptive analysis of the 3-item loneliness scale showed that higher proportions of female (21.4%, 4,087/19,054), older (20.4%, 1,066/5,219), Black/African/Caribbean/Black British or Other ethnicity CYP (18.5%, 173/933 and 19.1%, 100/524 respectively), having 3–4 siblings (19.7%, 980/4,976) and living in more deprived areas (e.g., with an IMD of 2) (18.5%, 1,024/5548) had more frequent experiences of loneliness throughout the pandemic (Table 3). For example, female CYP had a greater odds and risk of loneliness compared to males ([2.22 OR, 95%CI 2.08–2.38] [1.96 RR, 95%CI 1.85–2.08]) (Table 3). Those who reported having missed more than 10 days of schools had more frequent experiences of loneliness (25.2%, 248/983) in comparison to those who missed 1–10 days (20.3%, 717/3,531) or did not miss any days of school (16.8%, 2,203/13,079).

**Table 3:** Number of participants by demographic and frequency of loneliness, odds ratio and risk ratio of increased likelihood of more frequent experiences of loneliness, using the three-item loneliness scale.

	Number and proportion of participants experiencing loneliness		Unadjusted odds ratio analysis of being lonelier				Relative risk estimation of being lonelier			
	Lonelier n (%)	Same levels of loneliness/Less lonely n (%)	Odds ratio	P value	[95% confidence interval]		Risk ratio	P value	[95% confidence interval]	
**Age**	** **	** **	** **	** **	** **	** **	** **	** **	** **	** **
11	347 (11.83)	2,587 (88.17)	1.00				1.00			
12	483 (13.61)	3,065 (86.39)	1.17	0.03	1.01	1.36	1.15	0.03	1.01	1.31
13	643 (16.11)	3,348 (83.89)	1.43	<0.001	1.24	1.65	1.36	<0.001	1.21	1.54
14	778 (17.74)	3,608 (82.26)	1.61	<0.001	1.40	1.84	1.50	<0.001	1.33	1.69
15	1,023 (18.28)	4,573 (81.72)	1.67	<0.001	1.46	1.90	1.55	<0.001	1.38	1.73
16	1,055 (19.75)	4,287 (80.25)	1.83	<0.001	1.61	2.09	1.67	<0.001	1.49	1.87
17	1,066 (20.43)	4,153 (79.57)	1.91	<0.001	1.68	2.18	1.73	<0.001	1.54	1.93
**Sex at Birth**			** **	** **	** **	** **				
Female	4,087 (21.45)	14,967 (78.55)	2.22	<0.001	2.08	2.38	1.96	<0.001	1.85	2.08
Male	1,308 (10.93)	10,654 (89.07)	1.00				1.00			
**Region**			** **	** **	** **	** **				
East Midlands	388 (17.56)	1,822 (82.44)	1.00				1.00			
East of England	978 (16.17)	5,072 (83.83)	0.91	0.13	0.80	1.03	0.92	0.13	0.83	1.02
London	1,114 (18.10)	5,042 (81.90)	1.04	0.57	0.91	1.18	1.03	0.57	0.93	1.14
North East	207 (17.28)	0,991 (82.72)	0.98	0.84	0.81	1.18	0.98	0.84	0.84	1.15
North West	614 (17.02)	2,993 (82.98)	0.96	0.60	0.84	1.11	0.97	0.60	0.86	1.09
South East	901 (18.32)	4,016 (81.68)	1.05	0.44	0.92	1.20	1.04	0.44	0.94	1.16
South West	274 (18.10)	1,240 (81.90)	1.04	0.67	0.87	1.23	1.03	0.67	0.90	1.19
West Midlands	515 (16.98)	2,518 (83.02)	0.96	0.59	0.83	1.11	0.97	0.59	0.86	1.09
Yorkshire and The Humber	404 (17.33)	1,927 (82.67)	0.98	0.84	0.84	1.15	0.99	0.84	0.87	1.12
**Ethnicity**			** **	** **	** **	** **				
Asian/Asian British	796 (17.48)	3,758 (82.52)	1.01	0.75	0.93	1.10	1.01	0.75	0.94	1.08
Black/African/Caribbean/Black British	173 (18.54)	0,760 (81.46)	1.09	0.32	0.92	1.29	1.07	0.32	0.94	1.23
Mixed	284 (17.57)	1,332 (82.43)	1.02	0.77	0.89	1.16	1.02	0.77	0.91	1.13
Other	100 (19.08)	0,424 (80.92)	1.13	0.28	0.91	1.41	1.10	0.28	0.92	1.32
Prefer not to say	32 (16.93)	0,157 (83.07)	0.98	0.90	0.67	1.43	0.98	0.90	0.71	1.34
White	4,010 (17.28)	19,190 (82.72)	1.00				1.00			
**No. of Siblings**			** **	** **	** **	** **				
Only Child	435 (15.87)	2,306 (84.13)	1.00				1.00			
1–2 siblings	3,746 (16.98)	18,315 (83.02)	1.08	0.14	0.97	1.21	1.07	0.15	0.98	1.17
3–4 siblings	980 (19.69)	3,996 (80.31)	1.30	<0.001	1.15	1.47	1.24	<0.001	1.12	1.38
5 or more siblings	223 (19.29)	0,933 (80.71)	1.27	0.01	1.06	1.51	1.22	0.01	1.05	1.41
**IMD**			** **	** **	** **	** **				
1 (Most deprived)	906 (16.95)	4,439 (83.05)	0.99	0.85	0.90	1.09	0.99	0.85	0.92	1.07
2	1,024 (18.46)	4,524 (81.54)	1.10	0.04	1.00	1.20	1.08	0.04	1.00	1.16
3	1,034 (17.85)	4,760 (82.15)	1.05	0.24	0.96	1.15	1.05	0.24	0.97	1.13
4	1,121 (16.84)	5,536 (83.16)	0.98	0.71	0.90	1.07	0.99	0.71	0.92	1.06
5 (Least deprived)	1,310 (17.08)	6,362 (82.92)	1.00				1.00			

Higher proportions of those who reported OK or Very poor/poor physical health before the pandemic, were more likely to report feeling lonelier (20.7%, 1,302/6,280 and 21.7%, 121/558 by the 3-item loneliness scale) in comparison to those who reported Good/Very Good physical health (16.4%, 3,972/24,178). Those who reported OK mental health before the pandemic were more likely to report feeling lonelier (22.9%, 2,069/9,029) in comparison to those reporting Very Poor/Poor or Good/Very Good mental health pre-pandemic (19.3%, 523/2,706 and 14.5%, 2,803/19,281 by the 3-item loneliness scale). Of the 19,940 participants that reported a baseline health of 90 or above, 15.7% (3,122/19,940) reported feeling lonelier during the pandemic and of the 5,395 participants that described feeling lonelier, the highest reported pre-existing conditions were tiredness (50.4%), worrying (50.1%) and feeling depressed (35.5%).

At the time of testing, 74.5% (23,095/31,017) of the participants reported having no symptoms, 17% of whom felt lonelier during the pandemic according to the 3-item loneliness scale. 46.3% (3,670/7,921) of CYP who reported having between 1 and 21 symptoms stated that their symptoms were “Not very bad” or only “A little bad”, 14.5% (534/3,670) of whom felt lonelier during the pandemic. 30.2% (2,390/7,921) of CYP stated that their symptoms were “Quite bad”, 17.7% (424/2,390) of whom felt lonelier. 23.5% (1,861/7,921) of CYP stated that their symptoms were “Very bad” or “Extremely bad”, 25.4% (473/1,861) of whom felt lonelier. Using the Delphi definition of long COVID 6,982 participants were recorded as having met the criteria of the definition. Of those meeting this criterion, 34.0% (2,349/6,982) experienced loneliness more frequently during the pandemic.

Logistic regression of previous health, clinical vulnerability and pre-existing conditions showed that depression ([1.99 OR, 95%CI 1.87–2.12] [1.73 RR, 95%CI 1.65–1.82], base: No experience), worry ([1.94 OR, 95%CI 1.83–2.06] [1.72 RR, 95%CI 1.64–1.81], base: No experience) and a loss of interest ([1.84 OR, 95%CI 1.72–1.96] [1.63 RR, 95%CI 1.54–1.71], base: No experience) were the three pre-existing conditions most associated with increased odds and risk of more frequent experiences of loneliness (3-item loneliness scale) (Table 4). Those who reported their previous mental health as OK were also associated with increased odds and risk of more frequent experiences of loneliness ([1.24 OR, 95%CI 1.11–1.38] [1.19 RR, 95%CI 1.09–1.29], base: Very poor/Poor) in comparison to those who reported their previous mental health as Good/Very Good ([0.71 OR, 95%CI 0.64–0.79] [0.75 RR, CI% 0.69–0.82], base: Very poor/Poor).

**Table 4:** Number of participants by pre-existing condition and frequency of loneliness, odds ratio and risk ratio of increased likelihood of more frequent experiences of loneliness, using the three-item loneliness scale.

Just before the Covid-19 pandemic in early March 2020 were you experiencing. . . .	Lonelier			Same levels of loneliness / Less lonely			Unadjusted Odds ratio analysis of being lonelier				Relative risk estimation of being lonelier			
	Yes n (%)	No n (%)	Total n (%)	Yes n (%)	No n (%)	Total n (%)	Odds ratio	P value	[95% confidence interval]		Risk ratio	P value	[95% confidence interval]	
**Asthma**	683 (12.66)	4,712 (87.34)	5,395 (100.00)	2,823 (11.02)	22,798 (88.98)	25,621 (100.00)	1.17	0.00	1.07	1.28	1.14	<0.001	1.06	1.22
**Lung disease other than asthma**	19 (0.35)	5,376 (99.65)	5,395 (100.00)	69 (0.27)	25,552 (99.73)	25,621 (100.00)	1.31	0.30	0.79	2.18	1.24	0.29	0.83	1.85
**Allergy problems (skin eczema, hay fever, food allergies)**	1,753 (32.49)	3,642 (67.51)	5,395 (100.00)	7,517 (29.34)	18,104 (70.66)	25,621 (100.00)	1.16	<0.001	1.09	1.23	1.13	<0.001	1.07	1.19
**Problems with your stomach, gut, liver, kidneys or digestion**	331 (6.14)	5,064 (93.86)	5,395 (100.00)	1,222 (4.77)	24,399 (95.23)	25,621 (100.00)	1.31	<0.001	1.15	1.48	1.24	<0.001	1.12	1.37
**A neurological disease (one that affects the brain or nervous system e.g. epilepsy)**	70 (1.30)	5,325 (98.70)	5,395 (100.00)	340 (1.33)	25,281 (98.67)	25,621 (100.00)	0.98	0.86	0.75	1.27	0.98	0.86	0.79	1.22
**Any physical disability**	147 (2.72)	5,248 (97.28)	5,395 (100.00)	572 (2.23)	25,049 (97.77)	25,621 (100.00)	1.23	0.03	1.02	1.47	1.18	0.03	1.02	1.37
**Learning difficulties at school**	447 (8.29)	4,948 (91.71)	5,395 (100.00)	2,033 (7.93)	23,588 (92.07)	25,621 (100.00)	1.05	0.39	0.94	1.17	1.04	0.39	0.95	1.13
**Did you have an Educational Care and Health Plan (ECHP) giving extra support at school**	262 (4.86)	5,133 (95.14)	5,395 (100.00)	1,363 (5.32)	24,258 (94.68)	25,621 (100.00)	0.91	0.17	0.79	1.04	0.92	0.17	0.82	1.03
**Problems with your sleep, including getting to sleep, waking in the night or waking early**	1,251 (23.19)	4,144 (76.81)	5,395 (100.00)	4,156 (16.22)	21,465 (83.78)	25,621 (100.00)	1.56	<0.001	1.45	1.67	1.43	<0.001	1.35	1.51
**Problems with your eating including eating too much, eating too little or eating in an uncontrolled way (Binge eating)**	939 (17.41)	4,456 (82.59)	5,395 (100.00)	2,777 (10.84)	22,844 (89.16)	25,621 (100.00)	1.73	<0.001	1.60	1.88	1.55	<0.001	1.46	1.65
**A loss of interest or pleasure in doing things**	1,588 (29.43)	3,807 (70.57)	5,395 (100.00)	4,745 (18.52)	20,876 (81.48)	25,621 (100.00)	1.84	<0.001	1.72	1.96	1.63	<0.001	1.54	1.71
**Feeling down, depressed or hopeless**	1,914 (35.48)	3,481 (64.52)	5,395 (100.00)	5,553 (21.67)	20,068 (78.33)	25,621 (100.00)	1.99	<0.001	1.87	2.12	1.73	<0.001	1.65	1.82
**Worrying a lot about bad things or the future**	2,705 (50.14)	2,690 (49.86)	5,395 (100.00)	8,735 (34.09)	16,886 (65.91)	25,621 (100.00)	1.94	<0.001	1.83	2.06	1.72	<0.001	1.64	1.81
**Problems with headaches**	1,695 (31.42)	3,700 (68.58)	5,395 (100.00)	5,909 (23.06)	19,712 (76.94)	25,621 (100.00)	1.53	<0.001	1.43	1.63	1.41	<0.001	1.34	1.48
**Problems with tummy aches**	1,140 (21.13)	4,255 (78.87)	5,395 (100.00)	3,849 (15.02)	21,772 (84.98)	25,621 (100.00)	1.52	<0.001	1.41	1.63	1.40	<0.001	1.32	1.48
**Problems with friendships**	1,363 (25.26)	4,032 (74.74)	5,395 (100.00)	4,184 (16.33)	21,437 (83.67)	25,621 (100.00)	1.73	<0.001	1.62	1.86	1.55	<0.001	1.47	1.64
**Do you often feel very tired**	2,720 (50.42)	2,675 (49.58)	5,395 (100.00)	9,281 (36.22)	16,340 (63.78)	25,621 (100.00)	1.79	<0.001	1.69	1.90	1.61	<0.001	1.54	1.69
**Any other serious ill health**	133 (2.47)	5,262 (97.53)	5,395 (100.00)	462 (1.80)	25,159 (98.20)	25,621 (100.00)	1.38	0.00	1.13	1.67	1.29	0.00	1.11	1.50
**Smoking**	112 (2.08)	5,283 (97.92)	5,395 (100.00)	436 (1.70)	25,185 (98.30)	25,621 (100.00)	1.22	0.06	0.99	1.51	1.18	0.05	1.00	1.39
**Using e-cigarettes**	76 (1.41)	5,319 (98.59)	5,395 (100.00)	288 (1.12)	25,333 (98.88)	25,621 (100.00)	1.26	0.08	0.97	1.62	1.20	0.07	0.98	1.47

Logistic regression of COVID-19 and experienced symptoms showed that having a positive COVID-19 test result ([1.25 OR, 95% CI 1.18–1.33] [1.21 RR, 95%CI 1.15–1.27], base: negative test result) was associated with increased odds of reported loneliness. CYP that reported their symptom severity as “Very bad/extremely bad” ([2.00 OR, 95%CI 1.74–2.3] [1.75 RR, 95%CI 1.56–1.95], base: “Not very/a little”) and met the definition of having long COVID ([3.49 OR, 95%CI 3.28–3.72] [2.65 RR, 95%CI 2.53–2.78], base: not meeting the definition of long COVID) had a stronger association with more frequent experiences of loneliness.

Across all 5 categories of the EQ-5D-Y questionnaire those that reported having some problems had higher proportion (23% - 24%) of reported loneliness in comparison to those that reported no problems or a lot of problems in the 5 categories. Based on the SDQ, participants were evaluated as follows; 75.3% (23,349/31,015) “No impact”, 13.2% (4,091/31,015) “Minor impact” and 11.5% (3,575/31,015) “Definite impact”. The impact of the attributes was as follows; 68.9% (21,150/30,696) “Normal”, 10.5% (3,230/30,696) “Borderline” and 20.6% (6,316/30,696) “Abnormal”. Of those considered abnormal, 34% of participants felt lonelier, in comparison to those with normal or borderline attributes (13% and 29% respectively). Of the 6,316 who experienced abnormal impact 33% felt lonelier during the pandemic in comparison to those with normal or borderline reported impact (12% and 24% respectively). Using the 3-item loneliness scale, those with a SWEMBS metric score of between 9.51 and 17.43 were more likely to report feeling lonelier during the pandemic, with those having a score of 14.8 most likely to report feeling lonelier (44% of those with a score of 14.8).

## Discussion

### Principal findings

In this study we examined the experience of loneliness before and during the pandemic, and the associated influence of vulnerability and risk factors such as pre-existing physical and mental health conditions, ethnicity, age, sex at birth and index of multiple deprivation. Initial results demonstrated that before the pandemic a small minority of CYP often experienced loneliness.

### Comparison with other studies

When exploring the association between demographic factors and loneliness, this study confirmed previous research results that found older CYP, those assigned female at birth or were Black/African/Caribbean/Black British had more frequent experiences of loneliness throughout the pandemic [26]. Studies have shown that women and girls are more likely to have various sources of social support which will have been disrupted by the pandemic thereby contributing to their experience of loneliness [27]. Longstanding health inequalities affecting ethnic minorities that have been exacerbated by the pandemic are likely to be a factor in the increased frequency of loneliness experienced by the Black/African/Caribbean/Black British community [28].

During the pandemic, even when not in times of lockdown, clinically vulnerable people with pre-existing health conditions such as asthma, heart conditions and immunodeficient disorders were advised to shield at home for longer so as not to risk exposure to COVID-19. Clinically vulnerable CYP with pre-existing conditions had to stay at home for longer than their peers which might have enhanced their feelings of loneliness. While conditions such as these influenced their experience of loneliness, pre-existing conditions such as worry, tiredness and feeling depressed had the most effect. When looking at symptoms and outcomes due to COVID-19, those who recorded their symptoms as being most severe or having met the criteria of long COVID had more frequent experiences of loneliness. It may be that the severe impact or the constant destabilising effects of COVID caused CYP to take more time off school or attend/remain in hospital until they felt better. CYP having missed more than 10 days of school had a higher chance of experiencing more frequent episodes of loneliness. These potentially extended isolations from school and their peer groups may have enhanced their feelings of loneliness [29].

This study also showed that loneliness was experienced in a higher proportion of CYP in more deprived areas of England. While the lockdowns and restrictions hindered social development at a crucial age for all CYP, when lockdowns were eased and people had the opportunity to socialise again within certain limits (i.e. “bubbles”, only able to meet outside, only while wearing masks etc.), areas of higher deprivation perhaps had fewer resources and green spaces and therefore more restricted opportunities to socialise which may have been further impacted by higher crime rates and therefore issues with trust and socialisation in the community [30].

More notable changes in the experiences of loneliness during the pandemic were reported by those who said they had OK mental health before the pandemic in comparison to those that had poor or good mental health. This same pattern was observed from the use of the EQ-5D-Y questions for those with “some problems” pre-pandemic, having more frequent experiences of loneliness. This may be that those who reported very poor or poor health before the pandemic already had support systems in place, had developed inner resources and ways of identifying/ talking about ill health. Those who reported good or very good mental health, had fewer experiences of loneliness. Those who reported “OK” mental health before the pandemic may have benefited from support pre-pandemic. During the pandemic, however any concerns they had may have continued unchecked, exacerbating these concerns and potentially experiencing loneliness more often. A previous study explored the help-seeking behaviours of CYP in relation to mental health both before and during the pandemic and suggested that those who engaged with help seeking promotion tools were perhaps more open to mental health services [31].

While most CYP reported the same frequency of loneliness before and during the pandemic, similar proportions felt more or less lonely (17.4% and 18.9% respectively) during the pandemic. As the prevalence of increased frequency of loneliness was relatively low at 17.4% the odds ratios and risk ratios were similar when investigating the association between loneliness and factors such as health conditions and demographics. The feeling of loneliness during the pandemic may be influenced by the comparison between home life and school life. CYP who found school challenging or did not enjoy school may have benefited from learning from home, within their family bubbles, as they were not troubled by the challenges of stress or anxiety from difficulties with social interaction, bullying or exclusion, sensitivity to noisy environments, or academic challenge [32]. There may be individuals with an undiagnosed neurodivergent condition that found life easier to manage away from the stressors that school brings for them. However, schools play a vital role for CYP who have a disrupted, dissolved or abusive home life, either as a result of the pandemic or from longstanding stressors. However, the pandemic disrupted school life and such children were likely to experience increased feelings of loneliness and social isolation [33–35].

CYP with 3 or more siblings were identified as lonelier during the pandemic, which might seem counterintuitive given the greater opportunity for social interaction within a larger family unit. However, interactions between family members may have been more divided and siblings may have competed for parental affection, which may have been more in need as CYP were not able to see friends [36].

### Strengths and limitations

A clear strength of this paper and the CLoCk study as a whole, is the large sample size and sampling method. At the time of writing the CLoCk study is the largest national cohort study exploring long COVID in children and young people in the world. The breadth of knowledge gained from this study can ensure more comprehensive representation regarding post COVID health. We explored physiological, psychological and social factors in relation to loneliness. This may allow people to develop, seek and obtain preventive information and targeted interventions on how to maintain a better quality of life by having more control over their body and health [9].

A factor to consider when exploring loneliness of CYP during the pandemic is the role of social media. Social media provides a means of maintaining social relationships, virtual learning and health support through technology [37, 38]. However, participant experiences of insufficient relationships, isolation and bullying may be exacerbated by social media. It should also be noted that those who have limited access to social media may also feel isolated and be at greater risk of loneliness [37–39]. This study did not ask participants about their social media usage, so we were unable to examine this as a factor in loneliness.

A limitation of the study is that as a secondary analysis it is restricted to the variables initially collected. As this is a retrospective study, there is a risk of recall bias when asking CYP to remember how they felt and what they experienced many months prior to completing the survey [16]. Enrolment bias was noted with proportionally more girls than boys, and older CYP enrolling than were invited and a smaller proportion of CYP from more deprived areas enrolled than were invited into the study.

Selection bias may have had a further influence as disadvantaged groups may have struggled to access testing and so not been recorded in the PCR testing system. Finally, sampling bias could have been introduced as those with severe physical or mental health problems either before or during the pandemic may have chosen not to take part in the study while those who felt unaffected by the pandemic and did not feel that they had anything to contribute to the study may have refrained from participating. A further limitation of the study is that there is no threshold for the three-item and one-item loneliness scales above which someone is considered lonely and so cannot be standardised between studies [18].

Previous studies have explored the impact of the pandemic on the mental health of children and young people but there is limited information regarding the mental health of CYP during the pandemic that investigates both pre-existing physical and mental health conditions in the context of COVID-19, symptom severity and long COVID in relation to loneliness. This study confirmed associations between biological (such as sex at birth), social factors (such as days missed from school) and loneliness but, as highlighted by the biopsychosocial model, does not confirm that a factor definitively leads to loneliness therefore demonstrating the need for multi-faceted integrated approach when developing targeted interventions. To fully comprehend the impact of the pandemic over time it is important to follow up with the CYP involved at regular intervals to investigate the progression of their experience of loneliness.

### Future research

In order to more accurately evaluate loneliness, emotional responses, patterns of social contact and characteristics of social relationships need to be better understood and could be investigated in future studies. For example, someone may be unhappy due to the characteristics of their social relationships but may not consider themselves as lonely. While social factors such as social restrictions and days missed from school were explored, other factors such economic instability and social class would need to be further investigated to understand their impact on loneliness in depth. More research is needed into social network size, diversity and frequency of social contact to more comprehensively understand the role of social isolation and factors such as anxiety should be explored to understand the role of psychological factors.

## Conclusion

In conclusion we demonstrate associations between multiple factors and experiences of loneliness during the pandemic. There is a need for a multi-faceted integrated approach when developing interventions targeted at loneliness. Other factors such as economic instability, social class, social network size, diversity and frequency must be further researched to understand their impact on loneliness. It is important to follow up the CYP involved at regular intervals to investigate the progression of their experience of loneliness over time.

## Supporting information

S1 Appendix STROBE statement. Checklist of items that should be included in reports of observational studies.
(DOCX)

S1 Table Pre-pandemic loneliness table. Table of frequency of loneliness experienced before the pandemic against demographics, using the one-item loneliness scale.
(DOCX)

S2 Table Pandemic loneliness table. Table of frequency of loneliness experienced during the pandemic against demographics, using the one-item loneliness scale.
(DOCX)

S1 Fig Loneliness by demographics graph. Graph of proportion of loneliness experienced before and during the pandemic against demographics a) ethnicity, b) region, c) siblings and d) IMD, using the one-item loneliness scale.
(DOCX)
