# Supplementary material for: The CLoCk study: A retrospective exploration of loneliness in children and young people during the COVID-19 pandemic, in England
Source: PLoS One. 2023 Nov 21;18(11):e0294165. doi: 10.1371/journal.pone.0294165 (PMC10662715; doi:10.1371/journal.pone.0294165)
Supplement: S1 Table — Table of frequency of loneliness experienced before the pandemic against demographics, using the one-item loneliness scale. (DOCX) [file pone.0294165.s002.docx]

**S2 Table. Pre-pandemic loneliness table.** Table of frequency of loneliness experienced before the pandemic against demographics, using the one-item loneliness scale

|  | **Loneliness experienced before the pandemic n (%)** | | | | |
| --- | --- | --- | --- | --- | --- |
|  | **Never** | **Hardly ever** | **Occasionally** | **Some of the time** | **Often/Always** |
| **Age** |  |  |  |  |  |
| 11 | 1290 (44) | 940 (32) | 334 (11) | 291 (10) | 79 (3) |
| 12 | 1367 (39) | 1178 (33) | 441 (12) | 426 (12) | 136 (4) |
| 13 | 1406 (35) | 1310 (33) | 571 (14) | 532 (13) | 172 (4) |
| 14 | 1327 (30) | 1395 (32) | 745 (17) | 650 (15) | 269 (6) |
| 15 | 1357 (24) | 1646 (29) | 1097 (20) | 1075 (19) | 421 (8) |
| 16 | 1097 (21) | 1542 (29) | 1117 (21) | 1122 (21) | 464 (9) |
| 17 | 1026 (20) | 1467 (28) | 1136 (22) | 1125 (22) | 465 (9) |
| **Sex at Birth** |  |  |  |  |  |
| Female | 4090 (21) | 5640 (30) | 3901 (20) | 3911 (21) | 1512 (8) |
| Male | 4780 (40) | 3838 (32) | 1540 (13) | 1310 (11) | 494 (4) |
| **Ethnicity** |  |  |  |  |  |
| Asian/Asian British | 1396 (31) | 1374 (30) | 785 (17) | 744 (16) | 255 (6) |
| Black/African/Caribbean/Black British | 221 (24) | 266 (29) | 207 (22) | 171 (18) | 68 (7) |
| Mixed | 399 (25) | 494 (31) | 282 (17) | 305 (19) | 136 (8) |
| Other | 159 (30) | 146 (28) | 96 (18) | 86 (16) | 37 (7) |
| Prefer not to say | 75 (40) | 32 (17) | 33 (17) | 27 (14) | 22 (12) |
| White | 6620 (29) | 7166 (31) | 4038 (17) | 3888 (17) | 1488 (6) |
| **Region** |  |  |  |  |  |
| East Midlands | 620 (28) | 648 (29) | 423 (19) | 392 (18) | 127 (6) |
| East of England | 1790 (30) | 1884 (31) | 1028 (17) | 968 (16) | 380 (6) |
| London | 1727 (28) | 1925 (31) | 1110 (18) | 993 (16) | 401 (7) |
| North East | 355 (30) | 373 (31) | 212 (18) | 188 (16) | 70 (6) |
| North West | 1119 (31) | 1037 (29) | 629 (17) | 589 (16) | 233 (6) |
| South East | 1278 (26) | 1579 (32) | 856 (17) | 891 (18) | 313 (6) |
| South West | 390 (26) | 458 (30) | 280 (18) | 271 (18) | 115 (8) |
| West Midlands | 894 (29) | 902 (30) | 516 (17) | 523 (17) | 198 (7) |
| Yorkshire and The Humber | 697 (30) | 672 (29) | 387 (17) | 406 (17) | 169 (7) |
| **No. of Siblings** |  |  |  |  |  |
| Only Child | 779 (28) | 834 (30) | 493 (18) | 449 (16) | 186 (7) |
| 1-2 siblings | 6421 (29) | 6896 (31) | 3819 (17) | 3613 (16) | 1312 (6) |
| 3-4 siblings | 1359 (27) | 1425 (29) | 891 (18) | 924 (19) | 377 (8) |
| 5 or more siblings | 294 (25) | 301 (26) | 220 (19) | 222 (19) | 119 (10) |
| IMD |  |  |  |  |  |
| 1 Most deprived | 1573 (29) | 1469 (27) | 944 (18) | 935 (17) | 424 (8) |
| 2 | 1554 (28) | 1623 (29) | 1029 (19) | 952 (17) | 390 (7) |
| 3 | 1639 (28) | 1779 (31) | 982 (17) | 1008 (17) | 386 (7) |
| 4 | 1867 (28) | 2117 (32) | 1189 (18) | 1094 (16) | 390 (6) |
| 5 Least deprived | 2237 (29) | 2490 (32) | 1297 (17) | 1232 (16) | 416 (5) |
