# Supplementary material for: The CLoCk study: A retrospective exploration of loneliness in children and young people during the COVID-19 pandemic, in England
Source: PLoS One. 2023 Nov 21;18(11):e0294165. doi: 10.1371/journal.pone.0294165 (PMC10662715; doi:10.1371/journal.pone.0294165)
Supplement: S2 Table — Table of frequency of loneliness experienced during the pandemic against demographics, using the one-item loneliness scale. (DOCX) [file pone.0294165.s003.docx]

**S3 Table. Pandemic loneliness table.** Table of frequency of loneliness experienced during the pandemic against demographics, using the one-item loneliness scale

|  | **Loneliness experienced during the pandemic n (%)** | | | | |
| --- | --- | --- | --- | --- | --- |
|  | **Never** | **Hardly ever** | **Occasionally** | **Some of the time** | **Often/Always** |
| **Age** |  |  |  |  |  |
| 11 | 1367 (47) | 885 (30) | 291 (10) | 294 (10) | 97 (3) |
| 12 | 1451 (41) | 1068 (30) | 417 (12) | 441 (12) | 171 (5) |
| 13 | 1500 (38) | 1218 (31) | 495 (12) | 562 (14) | 216 (5) |
| 14 | 1414 (32) | 1263 (29) | 676 (15) | 707 (16) | 326 (7) |
| 15 | 1469 (26) | 1558 (28) | 982 (18) | 1104 (20) | 483 (9) |
| 16 | 1184 (22) | 1479 (28) | 942 (18) | 1174 (22) | 563 (11) |
| 17 | 1138 (22) | 1359 (26) | 990 (19) | 1193 (23) | 539 (10) |
| **Sex at Birth** |  |  |  |  |  |
| Female | 4444 (23) | 5210 (27) | 3428 (18) | 4080 (21) | 1892 (10) |
| Male | 5079 (42) | 3620 (30) | 1365 (11) | 1395 (12) | 503 (4) |
| **Ethnicity** |  |  |  |  |  |
| Asian/Asian British | 1536 (34) | 1260 (28) | 681 (15) | 742 (16) | 335 (7) |
| Black/African/Caribbean/Black British | 240 (26) | 273 (29) | 175 (19) | 169 (18) | 76 (8) |
| Mixed | 429 (27) | 464 (29) | 254 (16) | 330 (20) | 139 (9) |
| Other | 163 (31) | 129 (25) | 098 (19) | 93 (18) | 41 (8) |
| Prefer not to say | 78 (41) | 33 (17) | 27 (14) | 27 (14) | 24 (13) |
| White | 7077 (31) | 6671 (29) | 3558 (15) | 4114 (18) | 1780 (8) |
| **Region** |  |  |  |  |  |
| East Midlands | 683 (31) | 598 (27) | 355 (16) | 417 (19) | 157 (7) |
| East of England | 1926 (32) | 1778 (29) | 929 (15) | 981 (16) | 436 (7) |
| London | 1857 (30) | 1801 (29) | 980 (16) | 1061 (17) | 457 (7) |
| North East | 375 (31) | 333 (28) | 185 (15) | 214 (18) | 91 (8) |
| North West | 1161 (32) | 963 (27) | 548 (15) | 624 (17) | 311 (9) |
| South East | 1401 (28) | 1443 (29) | 780 (16) | 900 (18) | 393 (8) |
| South West | 422 (28) | 448 (30) | 243 (16) | 271 (18) | 130 (9) |
| West Midlands | 946 (31) | 875 (29) | 437 (14) | 547 (18) | 228 (8) |
| Yorkshire and The Humber | 752 (32) | 591 (25) | 336 (14) | 460 (20) | 192 (8) |
| **No. of Siblings** |  |  |  |  |  |
| Only Child | 859 (31) | 776 (28) | 400 (15) | 508 (19) | 198 (7) |
| 1-2 siblings | 6898 (31) | 6435 (29) | 3382 (15) | 3744 (17) | 1602 (7) |
| 3-4 siblings | 1427 (29) | 1337 (27) | 817 (16) | 953 (19) | 442 (9) |
| 5 or more siblings | 320 (28) | 257 (22) | 181 (16) | 253 (22) | 145 (13) |
| **IMD** |  |  |  |  |  |
| 1 Most deprived | 1690 (32) | 1349 (25) | 802 (15) | 989 (19) | 515 (10) |
| 2 | 1653 (30) | 1550 (28) | 850 (15) | 1031 (19) | 464 (8) |
| 3 | 1777 (31) | 1623 (28) | 924 (16) | 1011 (17) | 459 (8) |
| 4 | 1997 (30) | 2013 (30) | 1046 (16) | 1153 (17) | 448 (7) |
| 5 Least deprived | 2406 (31) | 2295 (30) | 1171 (15) | 1291 (17) | 509 (7) |
