# Supplementary material for: The CLoCk study: A retrospective exploration of loneliness in children and young people during the COVID-19 pandemic, in England
Source: PLoS One. 2023 Nov 21;18(11):e0294165. doi: 10.1371/journal.pone.0294165 (PMC10662715; doi:10.1371/journal.pone.0294165)
Supplement: S1 Fig — Graph of proportion of loneliness experienced before and during the pandemic against demographics a) ethnicity, b) region, c) siblings and d) IMD, using the one-item loneliness scale. (DOCX) [file pone.0294165.s004.docx]

**S4 Figure. Loneliness by demographics graph.** Graph of proportion of loneliness experienced before and during the pandemic against demographics a) ethnicity, b) region, c) siblings and d) IMD, using the one-item loneliness scale

a)

b)

c)

d)
